# Fertility analysis of intraspecific hybrids in *Vitis vinifera* and screening of superior hybrid combinations

**DOI:** 10.3389/fpls.2022.940540

**Published:** 2022-08-11

**Authors:** Zhi-Lei Wang, Fei Yao, Miao Hui, Dong Wu, Ying Wang, Xing Han, Xiao Cao, Yi-Han Li, Hua Li, Hua Wang

**Affiliations:** ^1^College of Enology, Northwest A&F University, Xianyang, China; ^2^Shaanxi Engineering Research Center for Viti-Viniculture, Xianyang, China; ^3^China Wine Industry Technology Institute, Yinchuan, China; ^4^Engineering Research Center for Viti-Viniculture, National Forestry and Grassland Administration, Xianyang, China

**Keywords:** *V. vinifera*, grape breeding, intraspecific hybridization, comprehensive fertility, hybrid compatibility

## Abstract

The correlations were explored between fertility indicators of intraspecific *V. vinifera* hybrids and different cultivars were subjected to selfing or used in reciprocal crosses by testing them as female parents or male parents. Two cold-resistant and four high-quality cultivars were selected, and the offspring of fourteen crosses and six self-combinations were evaluated. The pollen viability of the six cultivars was determined by TTC staining. Compatibility and the rates of fruit-setting, seediness, germination, emergence, and seedling were measured as parameters that can affect fertility of both hybridization and self-crossing processes. Using principal component analysis, the six fertility indexes were transformed into comprehensive principal components, and the weights of the indexes were determined. Combined with the membership function method, the fertility index was comprehensively evaluated for different crosses to screen for hybrid combinations with higher fertility. The results showed a high positive correlation between the pollen viability of the cultivar subjected to selfing and the fruit-setting rate, seediness rate, and hybrid compatibility index of the cultivar used as the male parent for crossing. Additionally, there was a one-to-one positive correlation between the fruit-setting rate, germination rate, emergence rate, and seedling rate of the selfed cultivar and the fruit-setting rate, germination rate, and seedling rate of the cultivar used as the female parent for crossing. There was some variation in the comprehensive fertility index values for the parents and combinations in different years. The comprehensive fertility index was always the highest for Ecolly as the male parent. The composite fertility index values were relatively high when Dunkelfelder, Cabernet Sauvignon, or Marselan were used as the female parent. The combinations of C1 (Cabernet Sauvignon × Ecolly), C3 (Marselan × Ecolly) and C6 (Dunkelfelder × Ecolly) exhibited relatively high comprehensive fertility indices, and pedigree clustering shows that these three combinations cluster into one class of highly fertile hybrid combinations. This study provides the basis for effective intraspecific hybrid breeding of grape (*V. vinifera*).

## Introduction

In nature, plants are constantly challenged by adverse abiotic environmental conditions such as drought, high temperature, cold, nutrient deficiencies, and excessive salinity or toxic metal levels in the soil. These abiotic stresses limit the use of arable land globally and negatively impact crop productivity (Zhang et al., 2021). Grape is the world’s most cultivated fruit, but low temperature stress significantly restricting the development of global viticulture, especially in northern China (Han et al., 2021), the Russian Far East (Nenko et al., 2016, 2019), the northeastern United States (Atucha et al., 2018; Schrader et al., 2019), and northeastern Canada (Fisher and Jamieson, 2000). Breeding new high-quality cold-resistant grape varieties adapted to unfavorable environmental conditions is crucial for the sustainable development of the global grape and wine industry (Wang Z. L. et al., 2021). It has been challenging for traditional interspecific hybrid breeding to breed new grape cultivars that are both high-quality and cold-resistant (Sun et al., 2011; Manso-Martínez et al., 2020). In addition, resistant cultivars bred by interspecific hybridization often contain higher amounts of allergens than cultivars of *V*. *vinifera*, which will limit the acceptance of such cultivars by consumers (Curioni et al., 2021). Thus, intraspecific recurrent selection in *V. vinifera* may be an effective method for high-quality and cold-resistant breeding of grapes. For this strategy to be successful, it is necessary to understand the correlation between different fertility indicators of intraspecific hybridization and to identify excellent intraspecific hybridization combinations.

The genetic relationships between grape cultivars are complex, with obvious differences in pollen fertility, hybrid compatibility and fertility (Luisa Badenes and Byrne, 2012). Previous work found obvious differences in pollen fertility among cultivars and effects of culture medium, but no relationship to changes in chromosome number (Wang L. et al., 2021). Affected by the degree of bud opening, the strength of pollen stigma receptivity can also differ for different combinations (Xin et al., 2019). The fruit-setting rate, the hybrid fruit seediness rate, and the average number of seeds per fruit were all positively correlated with selfing characteristics of the female parent and were strongly affected by the parthenocarpic ability of the female parent (Li et al., 2020). The mechanism of embryo abortion has been characterized from the morphological (Li, 2009; Pan et al., 2011; Liu et al., 2016), physiological and biochemical (Wang et al., 2004; Dong and Guo, 2008; Li et al., 2014; Yang et al., 2017; Iván et al., 2018), and molecular levels (Nicolas et al., 2013; Nwafor et al., 2014; Wang, 2017). Focusing on the molecular and genetic mechanisms controlling grape seed size, Nicolas et al. (2013),Wang et al., 2016, 2015, and Carolina et al. (2016) found that major differences identified during seed development were associated with hormone and epigenetic regulation, the development of the seed coat and endosperm, and the formation of seed identity complexes. Chai (2005) and Lin et al. (2009) compared the germination and seedling rates of cultivars *Vitis vinifera*, *Vitis labrusca*, and Franco-american, with *Vitis vinifera* and Franco-american cultivars showing higher and lower germination rates, respectively. Wang et al. (2022) also evaluated seed germination and seedling characteristics of *Vitis vinifera* cultivars and found significant variation, with higher germination rates of Ecolly, Garanior, and Marselan.

Previous research on the fertility of grape hybrids has mainly focused on pollen germination, stigma receptivity, tubular growth, embryo development, and fruit abortion mechanism during interspecific hybridization (Guifen et al., 2016; Zhao et al., 2017; Wang, 2018; Morimoto et al., 2019; Wang et al., 2022). Few studies have focused on potential differences within specific cultivars of *Vitis vinifera*, with little work to explore the complex relationship between intraspecific hybrid fertility indicators to identify suitable parents and screen for superior hybrid combinations. To facilitate application of intraspecific recurrent selection in *Vitis vinifera*, the goal of this work was to carry out fertility analysis of different hybrid combinations within cultivars of *Vitis vinifera* and to screen hybrid combinations with higher comprehensive fertility index values. Because traits can be autosomal or sex-linked, reciprocal crosses were performed. The results of this work should help identify appropriate materials for the breeding of new high-quality, cold-resistant grape cultivars.

In this study, six cultivars of *Vitis vinifera* were used as the test parents. The fruit-setting, seediness, germination, emergence, and seedling rates, as well as the compatibility index values were determined for the hybrid and selfing combinations. Correlation and principal component analysis were carried out on the fertility indicators, and the comprehensive fertility index values of the selfed cultivars and combinations were evaluated by the membership function method to provide a scientific basis for intraspecific hybridization.

## Materials and methods

### Materials and experimental field

With the primary research, the six wine grape cultivars of *V. vinifera* were used as experimental materials, including two high-quality cold resistant new wine grape cultivars (Meili, Ecolly) and four high-quality wine grape cultivars [Garanior (He, 2015), Dunkelfelder, Marselan (Zhan and Li, 2010), Cabernet Sauvignon]. The cultivars were selected from an experimental vineyard of the Northwest Agriculture and Forestry University (NWAFU) located in Yangling of Shanxi Province (lat. 34°N, long. 108°E), China. Self-rooted vines of *Vitis vinifera* L. were planted in 2013. Vine rows were oriented west—east, with vines spaced in 1.0 × 2.5 m rows. The vines were cordon-trained and pruned to two buds per spur. All viticultural practices were performed according to local standards. The characteristics of the six cultivars are presented as Supplementary Table 1.

### Experimental design

A cold-resistant wine grape cultivars, Ecolly or Meili, was used as one parent and four high-quality wine grape cultivars with relatively weak cold resistance were used in a reciprocal cross test design for a total of 14 hybrid combinations. Additionally, self-crossing was performed with the six cultivars. The hybridization and selfing combinations are presented as Supplementary Table 2. Samples (*n* = 6–20) were randomly selected and measured for each combination. The pollen viability and rates of fruit-setting, seediness, germination, emergence, and seedling were measured and analyzed in two consecutive years.

### Experimental methods

#### Pollen collection and determination of viability

Well-developed flower spikes were collected at the beginning of flowering. The pollen was rubbed out, evenly spread on a clean carton, and fully dried under a fluorescent lamp until the pollen was dispersed. After being filtered through a 120-mesh sieve, the pollen was fully ground with a mortar to break the pollen wall, and finally collected in a labeled centrifuge tube indicating the collection date and variety.

The pollen viability was determined using the 2,3,5-triphenyltetrazolium chloride (TTC) staining method (Fu and Geng, 2014). Briefly, the pollen was spread on a glass slide, a single drop of TTC solution was dropped on to the pollen and mixed evenly with a glass rod, the sample was covered with a cover glass, and then incubated in an incubator (35–40°C) for staining for 10–15 min. The samples were then observed under low magnification (10 × 10), and three visual fields were randomly selected with at least 100 pollen grains for each treatment. The numbers of viable (bright red) pollen and total pollen were counted, and average values were calculated.

#### Female parent emasculation and pollination cross

Hybridization experiments were carried out in May 2020 and May 2021 in an experimental vineyard of NWAFU. Emasculation and bagging isolation of grape inflorescences were performed 3 days before flowering. Pollination was done with a brush the next morning after emasculating, with three rounds of pollination on three consecutive days. After pollination, the inflorescences were bagged and the number of flowers was counted. The bagging was removed after 2–3 weeks to allow the fruit to develop naturally.

#### Measurement of fruit-setting rate, seediness rate, and compatibility index

Two months after hybrid pollination, the fruit-setting was determined. After the grapes had fully ripen, they were harvested and the seed setting of the fruit was measured. The compatibility index is equal to the ratio of the number of full mature seeds harvested to the total number of flowers that could have been pollinated.

#### Detection of germination rate, emergence rate and seedling rate

The seed germination and seedling raising experiments were carried out in the research greenhouse of NWAFU. With reference to the method of Wang et al. (2022) the seeds were subjected to germination, on-demand sowing (sowing according to the number of holes in the seedling tray), and transplanting experiments. The seeds were soaked in 1% sodium hypochlorite for 15 min before germination, and then fully coated with 25 g/L Syngenta fludioxonil seed coating. The sterilized seeds were spread evenly in a KangLi automatic intelligent sprout machine, incubated at a temperature from 28 to 38°C, and distilled water was added for 360° intermittent watering. The number of cracks or whitening were assessed every day as indicating germination. The germinated seeds were sown into a seedling tray. After the seeds emerged and two true leaves were unfolded, the number of seedlings was counted. The seedlings were then transplanted into 10 cm gallon pots (containing nutrient soil) for hardening. When the plants had grown to have four or five true leaves and reach a certain height, the numbers of survivors were counted and transplanted into the field for planting.

### Assay method of primary indicators

The pollen viability (%) equals the number of viable pollen divided by the total number of pollen × 100%. The fruit-setting rate (%) equals the number of fruit swelling divided by the number of flowers in the combination × 100%. The seediness rate (%) equals the number of fruits with fully mature seeds divided by the total number of fruits in the combination × 100%. The compatibility index equals the number of harvested full seeds divided by the number of flowers in combination. The germination rate (%) equals the total number of germinated seeds divided by the total number of seeds tested × 100%. The emergence rate (%) equals the number of seedlings (two pieces of cotyledon) divided by the number of seeds sown × 100%. The seedling rate (%) equals the number of surviving seedlings divided by the number of transplanted seedlings × 100%.

### Data processing

Evaluation of fertility is based on the evaluation of the various subordinative function indices in the form,


$$Uij=\frac{x_{ij}-x_{jmin}}{x_{jmax}-x_{jmin}}$$

(Positive correlation, including fruit-setting rate, seediness rate, compatible index, germination rate, emergence rate and seedling rate).

Here, *i* is a particular parent or combination, *j* is a particular index, *Xij* is the testing value of the index *j* of parent or combination *i*, *Xjmin* is the minimum value of index *j* for all parents or combinations, *Xjmax* is the maximum value of index *j* of all parents or combinations, *Uij* is the subordinative function value of parent or combination *i*, and index *j* that relates to fertility.

Microsoft Excel 2013 was used to record and process the original data and calculate the average and coefficient of variation. Analysis of variance and systematic cluster were performed in SPSS 22.0 statistical software. Heat Map with Dendrogram and Principal component analysis were performed using Origin 9.0 software. Weight coefficient analysis was performed using SPSSAU, an online platform for data analysis.^1^

## Results

### Fertility indexes

#### Identification of pollen viability

**FIGURE 1:**
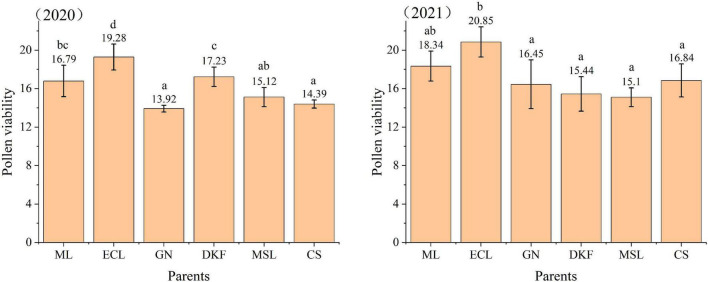
Pollen viability of six tested cultivars by TTC staining in 2020–2021. The data in this figure were tested by one-way ANOVA with Tuker’s test (*P* ≤ 0.05), and the same letter indicates that there is no significant difference in pollen viability among different parents. ML, ECL, GN, DKF, MSL, and CS represent cultivars of Meili, Ecolly, Garanior, Dunkelfelder, Marselan, and Cabernet Sauvignon.

The pollen viability was determined for the six tested cultivars by TTC staining, and the results are shown in Figure 1. There were significant differences in the pollen viability among different cultivars, and there were changes in the pollen viability of the tested parents in different years. The pollen viability of Ecolly was relatively stable and was the highest among the six tested parents both years. In 2020, the pollen viability of different parents was in the order of: Ecolly > Dunkelfelder > Meili > Marselan > Cabernet Sauvignon > Garanior. In 2021, the pollen viability of different parents was in the order of: Ecolly > Meili > Cabernet Sauvignon > Carnival > Dunkelfelder > Marselan.

#### Statistics on self-fertility

The fruit-setting rate, seediness rate, and compatibility index of the six tested parents were investigated and counted, and the results are shown in Table 1. Among the six self-crossing combination tested, the fruit-setting rates and seediness rates of Garanior were the highest for two consecutive years, 52.17 and 95.56, respectively, in 2020, 51.41 and 95.89, respectively, in 2021. Self-compatibility index values of Marselan were the highest for two consecutive years, 0.9398 and 0.8889 in 2020 and 2021, respectively. The average fruit-setting rate, average seediness rate, and average compatibility index value of the six self-crossing combination tested were 38.62, 89.76, and 0.59, respectively, in 2020 and were 36.84, 85.42, and 0.58, respectively, in 2021.

**TABLE 1:** Fruit-setting rate, seediness rate and compatibility index of selfing combinations.

Self-combinations	Number of flowers		Fruit-setting rate (%)		Seediness rate (%)		Compatibility index	
	
	2020	2021	2020	2021	2020	2021	2020	2021
C15 (ML)	789	669	23.95	25.11	90.48	89.29	0.3004	0.3498
C16 (ECL)	606	684	37.46	26.32	89.43	62.22	0.6188	0.6184
C17 (GN)	345	426	52.17	51.41	95.56	95.89	0.6783	0.6479
C18 (DKF)	465	480	36.13	39.38	85.12	84.13	0.4645	0.4938
C19 (MSL)	399	513	47.37	45.03	84.13	87.45	0.9398	0.8889
C20 (CS)	987	870	34.65	33.79	93.86	93.54	0.5289	0.5034
Average	598.50	607.00	38.62	36.84	89.76	85.42	0.59	0.58
Coefficient of variation / %	37.93	24.86	23.61	25.83	4.65	12.95	33.5	28.83

The germination, emergence, and seedling rates of the inbred seeds were investigated and counted, and the results are shown in Table 2. Among the self-cross combinations tested, the germination rate of Ecolly were the highest for two consecutive years, 71.53 and 96.90 in 2020 and 2021, respectively. The seedling rate of Meili was the highest for two consecutive years, 89.29 and 91.21 in 2020 and 2021, respectively. There were great differences for the different self-cross combinations. The germination rates were generally higher for Ecolly and Marselan, with seed germination rates of 76.80 and 74.40, respectively, in 2020, and 83.92 and 84.21, respectively, in2021. The germination rates were generally poor for Dunkelfelder and Cabernet Sauvignon, with seed germination rates of 10.19 and 9.77, respectively, in 2020, and 9.70 and 13.01, respectively, in 2021. The average germination rate, average emergence rate, and average seedling rate of the six self-cross combinations tested in 2020 were 47.14, 57.45, and 86.06, respectively. The average germination rate, average emergence rate and average seedling rate of the six self-cross combinations tested in 2021 were 56.24, 60.33, and 83.81, respectively.

**TABLE 2:** Germination rate, emergence rate and seedling rate of self-inbred seeds.

Self-combinations	Number of flowers		Fruit-setting rate (%)		Seediness rate (%)		Seedling rate (%)	
	
	2020	2021	2020	2021	2020	2021	2020	2021
C15 (ML)	237	234	56.12	67.95	42.11	57.23	89.29	91.21
C16 (ECL)	375	423	76.80	83.92	71.53	96.90	87.38	90.70
C17 (GN)	234	276	55.56	78.62	61.54	61.29	85.00	81.95
C18 (DKF)	216	237	10.19	9.70	59.09	47.83	84.62	81.82
C19 (MSL)	375	456	74.40	84.21	47.67	53.13	85.71	72.55
C20 (CS)	522	438	9.77	13.01	62.75	45.61	84.38	84.62
Average	326.50	344.00	47.14	56.24	57.45	60.33	86.06	83.81
Coefficient of variation / %	33.48	28.03	58.33	57.26	17.07	28.49	2.03	7.50

#### Statistics on hybrid fertility

The fruit-setting rate, seediness rate, and compatibility index of the six tested parents were investigated and counted, and the results are shown in Table 3. Among the 14 hybrid combinations tested, the seeding rates were the highest in both years for combination 6, 65.63 and 75.93 in 2020 and 2021, respectively. There were great differences in fruit-setting rate among the different combinations tested, with generally better fruit-setting rates for C1, C2, C3, C4, C6, and C7. In 2020, the fruit-setting rates of C1, C2, C3, C4, C6, and C7 were 24.27, 18.55, 21.37, 19.40, 17.56, and 15.75, respectively, and in 2021, the rates were 16.49, 24.75, 18.51, 16.67, 19.08, and 18.70, respectively. There were great differences in the hybridization compatibility index values among different tested combinations, with better values for C3 and C6. In 2020, the hybrid compatibility index values of C3 and C6 were 0.2502 and 0.2843, respectively, and in 2021, the values were 0.2833 and 0.4452, respectively. The average fruit-setting rate, average seediness rate, and average hybrid compatibility index value of the 14 hybrid combinations tested were 15.22, 18.51, and 0.08, respectively, in 2020 and were 14.77, 19.10, and 0.09, respectively, in 2021.

**TABLE 3:** Fruit-setting rate, seediness rate and compatibility index of hybrid combinations.

Cross combination (♀ × ♂)	Number of pollinated flowers		Fruit-setting rate (%)		Seediness rate (%)		Hybrid compatibility index	
	
	2020	2021	2020	2021	2020	2021	2020	2021
C1 (CS × ECL)	1,582	1,746	24.27	16.49	1.04	84.72	0.0076	0.3895
C2 (MSL × ML)	1,391	998	18.55	24.75	6.59	1.62	0.0295	0.0050
C3 (MSL × ECL)	1,151	886	21.37	18.51	35.77	68.29	0.2502	0.2833
C4 (CS × ML)	2,134	1,728	19.40	16.67	2.17	2.43	0.0098	0.0104
C5 (DKF × ML)	1,115	919	16.23	13.71	56.35	11.11	0.3049	0.0381
C6 (DKF × ECL)	911	849	17.56	19.08	65.63	75.93	0.2843	0.4452
C7 (ECL × CS)	1,924	1,717	15.75	18.70	21.78	3.12	0.0743	0.0157
C8 (ECL × GN)	1,026	1,189	12.28	10.09	14.29	4.17	0.0419	0.0109
C9 (ECL × MSL)	1,649	1,646	14.19	12.58	11.11	9.66	0.0358	0.0219
C10 (ECL × DKF)	2,164	1,708	11.92	14.58	10.08	4.82	0.0305	0.0152
C11 (ML × GN)	1,984	2,147	10.03	12.58	9.05	0.74	0.0192	0.0009
C12 (ML × CS)	2,139	2,944	10.99	8.32	11.06	0.00	0.0290	0.0000
C13 (ML × MSL)	1,786	1,917	10.58	13.15	9.52	0.79	0.0230	0.0010
C14 (ML × DKF)	1,893	2,419	9.98	7.57	4.76	0.00	0.0132	0.0000
Average	1632.07	1629.50	15.22	14.77	18.51	19.10	0.08	0.09
Coefficient of variation / %	26.10	36.67	28.83	30.43	104.45	158.16	131.03	168.91

The germination, emergence, and seedling rates of the hybrid seeds were investigated and counted, and the results are shown in Table 4. There were great differences for the different crosses. The germination rates were generally higher for C2, C3, and C7, with seed germination rates of 78.21, 78.98, and 77.62, respectively, in 2020, and 80.00, 83.24, and 77.55, respectively, in 2021. The emergence rates of C6, C7, C9, and C10 were generally higher: 94.44, 72.97, 87.80, and 80.00, respectively, in 2020, and 76.53, 85.53, 90.91, and 87.04, respectively, in 2021. The seedling rates were generally higher for C3, C6, C7, C9, and C10, with values of 85.71, 85.29, 85.19, 83.33, and 85.00, respectively, in 2020, and 84.31, 82.67, 83.08, 86.67, and 85.11, respectively, in 2021. The average germination rate, average emergence rate, and average seedling rate of the 14 hybrid combinations tested in 2020 were 59.93, 65.23, and 85.74, respectively. The average germination rate, average emergence rate and average seedling rate of the 14 hybrid combinations tested in 2021 were 64.98, 78.15, and 77.76, respectively.

**TABLE 4:** Germination rate, emergence rate and seedling rate of hybrid seeds.

Cross combinations (♀ × ♂)	Number of tested seeds		Germination rate (%)		Emergence rate (%)		Seedling rate (%)	
	
	2020	2021	2020	2021	2020	2021	2020	2021
C1 (CS × ECL)	48	1,155	66.67	85.45	68.75	75.08	77.27	90.15
C2 (MSL × ML)	78	5	78.21	80.00	62.30	75.00	84.21	66.67
C3 (MSL × ECL)	314	734	78.98	83.24	50.81	81.34	85.71	84.31
C4 (CS × ML)	70	18	28.57	50.00	85.00	55.56	88.24	60.00
C5 (DKF × ML)	697	130	11.76	25.38	54.88	54.55	84.44	72.22
C6 (DKF × ECL)	259	378	13.90	25.93	94.44	76.53	85.29	82.67
C7 (ECL × CS)	143	98	77.62	77.55	72.97	85.53	85.19	83.08
C8 (ECL × GN)	109	13	65.14	69.23	39.44	100	85.71	66.67
C9 (ECL × MSL)	65	36	63.08	91.67	87.80	90.91	83.33	86.67
C10 (ECL × DKF)	66	88	75.76	61.36	80.00	87.04	85.00	85.11
C11 (ML × GN)	79	2	58.23	0	54.35	0	88.00	0
C12 (ML × CS)	62	0	70.97	0	52.27	0	86.96	0
C13 (ML × MSL)	54	2	70.37	0	55.26	0	95.24	0
C14 (ML × DKF)	64	0	79.69	0	54.90	0	85.71	0
Average	150.57	265.50	59.93	64.98	65.23	78.15	85.74	77.76
Coefficient of variation / %	113.01	138.23	38.34	35.08	24.33	17.51	4.25	12.69

### Fertility indexes analysis

#### Correlation analysis of fertility indexes

**FIGURE 2:**
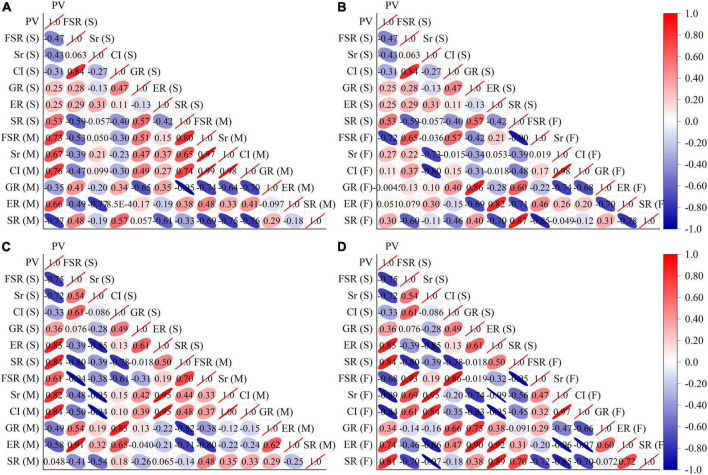
Correlation analysis of fertility indexes. **(A)** Shows the results of the correlation analysis between the fertility of selfing and the fertility of hybrid male parents for the experiments performed in 2020. **(B)** Shows the results of the correlation analysis between the fertility of selfing and the fertility of the hybrid female parent in 2020. **(C)** Shows the results of the correlation analysis between the fertility of selfing and the fertility of hybrid male parents in 2021. **(D)** Shows the results of the correlation analysis between the fertility of selfing and the fertility of the hybrid female parent in 2021. PV, FSR, Sr, CI, GR, ER, and SR represent fertility indexes of pollen viability, fruit-setting rate, seediness rate, compatibility index, germination rate, emergence rate, and seedling rate. (s) Cultivar as selfed, (M) Cultivars as male parent, (F) Cultivars as female parent.

As shown in Figure 2, correlation analysis of the fertility indicators for two consecutive years revealed a high positive correlation between the pollen viability of the tested cultivars and the fruit-setting rate, seediness rate and compatibility index of the hybrid male parent. Additionally, the analysis shows a high positive correlation between the fruit-setting rate obtained by selfing and the self-compatibility index. There was a high positive correlation between the self-bred seedling rate of a cultivar and the fruit-setting rate when the cultivar was used as the hybrid male parent. There was a high positive correlation between the fruit-setting rate of selfed cultivars and the fruit-setting rate of the hybrid female parent. There was a high positive correlation between the germination rate of a cultivar with the germination rate when that cultivar was used as the hybrid female parent. There was a high positive correlation between the seedling rate for selfing and the seedling rate when used as a hybrid female parent. There was a high negative correlation between the seedling rate of the cultivar subjected to selfing and the germination rate of the cultivar when used as the hybrid male parent. There was a high negative correlation between the pollen viability of each selfed cultivar and the fruit-setting rate of the hybrid female parent. There was a high negative correlation between the seedling rate of the selfed cultivar and the fruit-setting rate when the hybrid was used as the hybrid female parent. There was a high negative correlation between the fruit-setting rate and the seedling rate in a hybrid female parent, as well as a high negative correlation between the compatibility index and the germination rate of the hybrid female parent.

#### Principal component analysis of fertility indexes

**FIGURE 3:**
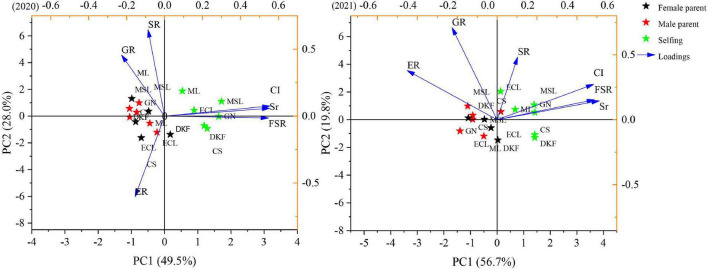
Principal component analysis of fertility indexes in 2020–2021. FSR, Sr, CI, GR, ER, and SR represent fertility indexes of fruit-setting rate, seediness rate, compatibility index, germination rate, emergence rate, and seedling rate.

Principal component analysis of the six fertility indicators was carried out with groups for each cultivar as the male parent, as the female parent, or selfed, and the results are shown in Figure 3. The tested crosses were clearly distinguished from the selfings, but there were less differences for crosses in which the cultivars were used in turn as the female parent and the male parent. The cumulative contribution rate of the two principal components in 2020 was 77.5%, so these two components effectively reflect most of the information on comprehensive fertility. The variance contribution rate of the first component was 49.5%, and the positive value of the load was the fruit-setting rate, the seediness rate, and the compatibility index. The variance contribution rate of the second component was 28.0%, the germination rate and seedling rate were larger in positive load values, and the load value in the emergence rate was larger in the negative direction. The cumulative contribution rate of the two principal components in 2021 was 76.5%, to again effectively reflect most of the information on comprehensive fertility. The variance contribution rate of the first component was 56.7%. The fruit-setting rate, the seeding rate, and the compatibility index had larger positive load values, and the germination rate and the seedling emergence rate had larger negative load values. The variance contribution rate of the second component was 19.8%, and the germination rate, seedling emergence rate, and seedling formation rate had larger positive load values.

From the load and contribution value of each index determined in the principal component analysis, the effects of the fertility index on the comprehensive fertility of the tested hybrid and selfing combinations were calculated, and the weights were determined. The results are presented as Supplementary Table 3. In 2020, the effects on the comprehensive fertility of the tested hybrid and selfing combinations from strong to weak were: compatibility index, seediness rate, fruit-setting rate, emergence rate, germination rate, and seedling rate. In 2021, the effects from strong to weak were: emergence rate, compatibility index, seediness rate, fruit-setting rate, germination rate and seedling rate.

### Membership function method for comprehensive evaluation of fertility

#### Comprehensive evaluation of the fertility of the tested cultivars

**FIGURE 4:**
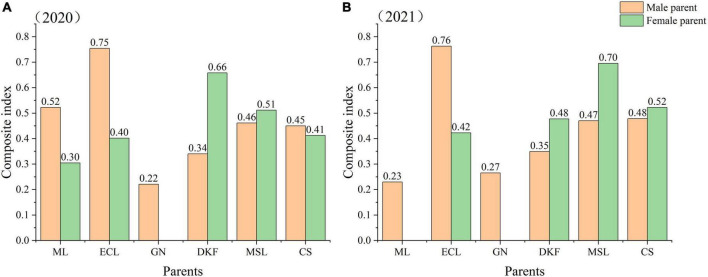
The comprehensive index of fertility of the tested parents as male or female parents in 2020–2021. Note: ML, ECL, GN, DKF, MSL, and CS represent cultivars of Meili, Ecolly, Garanior, Dunkelfelder, Marselan, and Cabernet Sauvignon. Since the flowering period of GN began first, in the experiment at the same site, GN could only be selected as a hybrid male parent. In 2021, the hybrid combination with ML as the female parent hardly harvested full seeds, which may be related to the premature emasculation. In order not to affect the authenticity of the overall results, the crosses with ML as the female parent were excluded from the analysis.

To comprehensively evaluate the fertility of the tested hybrid combinations, it is necessary to combine the contribution rates of the principal components and coordinate the fertility indicators of each principal component. The fertility indicators for cultivars used as male parent or female parent were standardized and quantified by the membership function method. These index values were then multiplied by the membership value and weight of each index and then summed to obtain the comprehensive index of fertility of each parent. The results are shown in Figure 4. The results for two consecutive years show a better comprehensive fertility index for use of Ecolly as the male parent than as the female parent. The fertility composite index values for use of Dunkelfelder or Marselan were better when used as the female parent than as the male parent. The composite fertility index values were very similar for using Cabernet Sauvignon as either the male or female parent in both years. In 2020, the comprehensive fertility index values of the six tested cultivars as male parents were in order from high to low: Ecolly, Meili, Marselan, Cabernet Sauvignon, Dunkelfelder, and Garanior. In 2020, the comprehensive fertility index values of the five tested hybrid parents as the female parent were in order from high to low: Dunkelfelder, Marselan, Cabernet Sauvignon, Ecolly, and Meili. In 2021, the comprehensive fertility index values of the six tested hybrid parents as male parents were from high to low: Ecolly, Cabernet Sauvignon, Marselan, Dunkelfelder, Garanior, and Meili. In 2021, the comprehensive fertility index values of the four tested hybrid parents as the female parent were from high to low: Marselan, Cabernet Sauvignon, Dunkelfelder, and Ecolly.

#### Comprehensive evaluation of the fertility of the tested hybrid combinations

**FIGURE 5:**
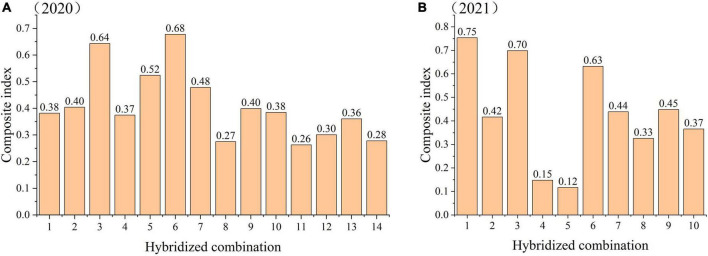
The comprehensive index of fertility of the tested hybrid combinations in 2020–2021. The hybrid combination with Meili as the female parent hardly harvested full seeds in 2021, which may be related to the premature emasculation. In order not to affect the authenticity of the overall results, Combinations 11, 12, 13, and 14 were removed from the analysis in 2021.

The fertility indicators of the 14 hybrid combinations tested were standardized and quantified by the membership function method. These index values were then multiplied by the membership value and weight of each index and then summed to obtain the comprehensive index of fertility of each parent. The results are shown in Figure 5. The results for two consecutive years showed that relatively high and stable composite fertility index performance of C3 and C6. The composite fertility index of C8 was relatively low and stable. The composite fertility index of C4 and C5 changed the most between 2020 and 2021. The comprehensive fertility indices determined in 2020 for the 14 tested hybrid combinations were from high to low: C6, C3, C5, C7, C2, C9, C10, C1, C4, C13, C12, C14, C8, and C11. In 2021, the order from high to low was C1, C3, C6, C9, C7, C2. C10, C8, C4, and C5.

#### Comprehensive analysis of hybrid fertility

**FIGURE 6:**
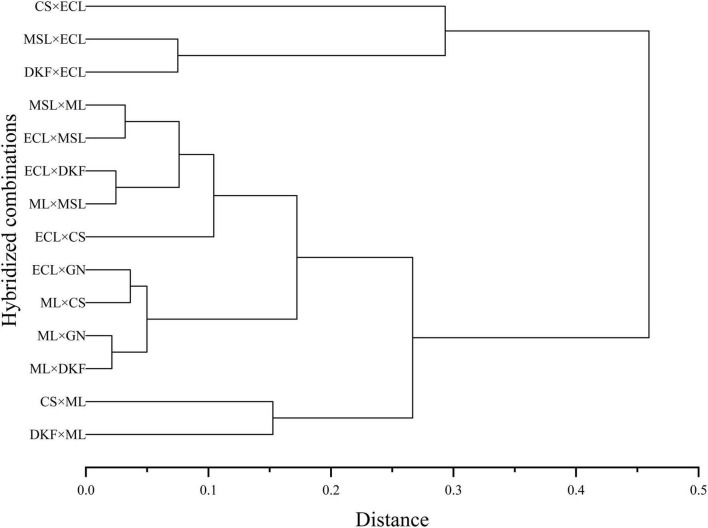
Systematic cluster analysis diagram of the comprehensive index of fertility of the tested hybrid combinations. For the hybrid combination with Meili as the female parent, only the comprehensive fertility index in 2020 was used for analysis.

Systematic cluster analysis was carried out on the comprehensive fertility index values of the 14 tested hybrid combinations for two consecutive years, and the results are shown in Figure 6. The tested combinations were grouped into three categories. C1 (CS × ECL), C3 (MSL × ECL), and C6 (DKF × ECL) were clustered into one category of highly fertile hybrid combinations. C2 (MSL × ML), C9 (ECL × MSL), C7 (ECL × CS), C10 (ECL × DKF), and C13 (ML × MSL) were clustered into one category and are moderately fertile hybrids. C4 (CS × ML), C11 (ML × GN), C14 (ML × DKF), C5 (DKF × ML), C8 (ECL × GN), and C12 (ML × CS) were clustered as one category of weak hybrid combinations.

## Discussion

### Fertility status of *Vitis vinifera*

Fruit-setting rate is the fertility basis of cross-breeding (Li et al., 2020). For both the inbred and hybrid combinations, fruit-setting rates were generally good, the coefficient of variation is relatively stable, and there was relatively small fluctuation of the average fruit-setting rate in different years. This likely reflects our choice of species, as grapes are closed-flowering pollinators and are highly self-compatible (Zhang, 2000). In addition, the parents we selected for testing were all cultivars of *V. vinifera*, with highly compatible intraspecific hybrids (Shymanovich et al., 2017). Compatibility is a main factor affecting breeding efficiency, and compatibility barriers can be pre-fertilization or post-fertilization barriers (Li et al., 2016). Pre-fertilization barriers include pollen that is unable to stick, germinate, and grow on the stigma, and the production of a large amount of callose, evident by low fruit-setting rate. Obstacles after fertilization are evident as hybrid embryos, abnormal development of endosperm, low viability or deformity of hybrids, and low seeding rate. In this study, for both inbred and hybrid combinations, there were significant variations in seeding rate and the compatibility index between years and combinations. In 2020, the coefficients of variation of seediness rate and compatibility index were 104.45 and 131.03%, respectively, and these values were even higher in 2021, 158.16 and 168.91%, respectively. The relatively stable fruit-setting rate indicated that obstacles before fertilization had little effect on the compatibility of the tested combinations, and the seediness rate was the main factor determining the compatibility of grape hybrids. This may reflect genomic sequence differences, genome rearrangement, epigenetic remodeling, maternal and paternal genome imbalance, or endosperm -imprinted genes (Bushell et al., 2003).

Temperature and humidity are key environmental conditions that affect seed germination (Kawano et al., 2020). In this study, the germination rates of the inbred parents, Ecolly and Marselan, were consistently higher than those of the other cultivars, and the germination rates of Dunkelfelder and Cabernet Sauvignon were always lower. The study conditions were carefully controlled with regularly spraying and constant temperature, so the results should not include effects of temperature and humidity. The characteristics of the seeds, such as seed size and seed coat thickness may be the most significant determinants of germination rate (Jisha et al., 2013; Wang et al., 2022). Ecolly and Marselan seeds are smaller and have a relatively thin seed coat, allowing the embryo to more easily break through the seed coat and complete the germination process. The seeds of Dunkelfelder and Cabernet Sauvignon are medium in size, and the seed coat is relatively hard, so it is difficult for the seed embryo to break through the hard seed coat for germination. Although Meili seeds also have a hard seed coat, they still maintain a moderate germination rate, which may be influenced by other factors. Studies have shown that the 1,000-grain weight of seeds is also the main determinant of seed germination (Jisha et al., 2013; Li et al., 2020), and characteristics such as seed maturity, endogenous hormone content, water content, dormancy type, and chilling requirements to break dormancy may also affect the germination characteristics of seeds (Walck et al., 2005; Pan et al., 2007; Gan et al., 2009; Daisuke et al., 2020). Germination of seeds and growth of seedlings are the beginning stages of the plant life cycle. In this study, the seedling emergence rate was highest for the inbred parent Ecolly in both years, which is consistent with our previous research results. The cultivars with higher germination vigor and germination rate also show a higher level of emergence rate. Our previous research results showed Ecolly had the highest germination among the six tested parents (Wang et al., 2022). Seedling rate is also a fertility indicator and has the lowest coefficient of variation for both inbred and hybrid combinations, which may be related to the seed dressing treatment. Studies have shown that seed dressing treatment may be able to control seedling blight (Ma et al., 2014).

### Factors affecting fertility of *Vitis vinifera*

Fertility is the key to the success or failure of plant hybrid breeding. In this study, hybrid populations were harvested from all 14 hybrid combinations, with clear differences in different fertility indicators. The comprehensive fertility index values ranged from 0.12 to 0.75, which facilitated screening of combinations with higher fertility. The correlation analysis showed a high positive correlation between the pollen viability of the cultivars and the fruit-setting rate, seediness rate and compatibility index of the hybrid male parent, consistent with previous research results (Guo, 2019). Studies have shown that the pollination period of cross-breeding and the seed-setting ability of the female parent significantly affect the fruit-setting rate and compatibility index of the cross (Li et al., 2020). Selecting a male parent with strong pollen viability is required to ensure the success of cross-breeding. Among the six tested cultivars in this study, the pollen viability of Ecolly was the highest for two consecutive years, indicating that Ecolly was the most suitable as the hybrid male parent. In addition, we found a one-to-one positive correlation between the fruit-setting rate, germination rate, emergence rate and seedling rate of the selfed cultivar and the fruit-setting rate, germination rate, emergence rate and seedling rate of the cultivar when used as the female parent. This is consistent with previous research results (Li et al., 2020). The germination and seedling characteristics of hybrid seeds can be greatly affected by genetic mechanisms of the female parent, so hybridization strategies that select a female parent with strong self-fertility are more likely to succeed (Zhuang, 2018). Considering the combined effects of the fertility indicators of the female parent, the results show that Dunkelfelder, Marselan, and Cabernet Sauvignon are the most suitable for use as female parents.

Grape is a self-pollinating plant, and the effect of self-fertility is much better than that of cross-fertility (Zhang, 2000). In this study, the principal components analysis clearly distinguished the self-crossing from the cross, but it was difficult to distinguish the parents of the hybrid as the male and female parents. The weight of the fertility index is calculated according to the load and contribution value of the fertility index of different combinations of parents. There were changes between years, but overall, the seediness rate and the compatibility index have the largest weights, indicating that whether the hybrid fruit has plump seeds plays a key role in hybrid fertility. In practical operation, we found that using Meili as the male parent, many large and small seeds appeared in the hybrid fruits. When the seeds of these fully mature fruits were separated, few seeds were able to reach a full state. This incompatibility allows setting, but not germination, due to abortive seeds during later stages of seed development. Studies of the mechanisms responsible for abortion suggest that the control of seed size and weight may involve multiple layers of regulation of embryo, endosperm, and seed coat (Wei et al., 2021). These three structures have been the focus of studies of embryogenesis (Lau et al., 2012), endosperm development (Olsen, 2020; Dai et al., 2021), seed coat formation (Figueiredo and Köhler, 2014; Khan et al., 2014), and control of seed size (Kesavan et al., 2013; Orozco-Arroyo et al., 2015). However, ovary enlargement does not represent embryo development, and the factors affecting hybrid fertility are complex and diverse, so the evaluation of a single index often produces one-sided or contradictory results (Pan et al., 2018). Thus, when customizing the combination of crosses, a male parent with higher pollen viability should be matched with a female parent with high self-seeding rate, more seeds per fruit, and relatively high germination and seedling emergence rates. In this way, more hybrid populations can be obtained for improved efficiency of breeding.

## Conclusion

The results show that parental pollen vigor was closely related to the fruit-setting rate, seediness rate, and compatibility index of the hybrid male parent. Grape cultivars with higher pollen viability have relatively high fruit-setting rate, seediness rate, and compatibility index as the hybrid male parent. Among the six tested parents, Ecolly exhibited the highest pollen viability for two consecutive years, suggesting this cultivar should be preferentially selected as the hybrid male parent. The genotype of the parents had a high positive correlation with the fertility of the hybrid seed. For the cultivars with higher fruit-setting rate, germination rate, emergence rate and seedling rate for selfing, the fruit-setting rate, germination rate, emergence rate and seedling rate were high when used as the female parent for crosses. Considering the effects of fruit-setting rate, seediness rate, compatibility index, germination rate, emergence rate and seedling rate, Dunkelfelder, Cabernet Sauvignon, and Marselan can be preferentially used as female parents for hybridization. Systematic cluster analysis was carried out on the comprehensive fertility index of 14 tested hybrid combinations for two consecutive years. C1 (CS × ECL), C3 (MSL × ECL), and C6 (DKF × ECL) were identified as high fertility hybrid combinations that should be preferably selected.

The purpose of this study was to guide intraspecific hybridization in *V. vinifera* to obtain more hybrid populations. Next steps will be to explore the physiological, biochemical, and molecular mechanisms underlying the factors that cause fertility. Future work should also further explore the abortive process that occurs in intraspecific hybridization, in which fruit is produced but germination does not occur.

## Data availability statement

The original contributions presented in this study are included in the article/Supplementary material, further inquiries can be directed to the corresponding authors.

## Author contributions

Z-LW, FY, HW, and HL: conceptualization. Z-LW, YW, XC, and DW: data curation. MH and XH: formal analysis. HW and HL: funding acquisition and project administration. FY and Y-HL: methodology. All authors have read and agreed to the published version of the manuscript.

## Footnotes

1
https://spssau.com
