# Supplementary material for: Fertility analysis of intraspecific hybrids in Vitis vinifera and screening of superior hybrid combinations
Source: Front Plant Sci. 2022 Aug 11;13:940540. doi: 10.3389/fpls.2022.940540 (PMC9403984; doi:10.3389/fpls.2022.940540)
Supplement: Supplementary file 1 [file Data_Sheet_1.zip › Supplementary Material/Table 1.docx]

**Table S1 Varietal characteristics and seed characteristics of the tested cultivars**

| Cultivars | Flowering stage | Characteristics | Seed Characteristics |
| --- | --- | --- | --- |
| Meili (ML)  (Zhang, 2000) | 5.22～6.1 | Red mid-variety with strong cold resistance. | Large seed, seed coat is medium thickness. Each fruit contains 1～3 seeds. |
| Ecolly (ECL)  (Zhang, 2000) | 5.13～5.29 | White mid-variety with strong cold resistance. | Small seed, seed coat is relatively thin. Each fruit contains 1～3 seeds. |
| Garanior (GN)  (He, 2015) | 5.9～5.26 | High-quality, red early-maturing variety with poor cold resistance. | Small seed, seed coat is relatively thin. Each fruit contains 2～4 seeds. |
| Dunkelfelder (DKF)  (Zhan and Li, 2010) | 5.13～5.29 | High-quality, red mid-variety with medium cold resistance. | Medium-sized seed, seed coat is relatively thin. Each fruit contains 1～4 seeds. |
| Marselan (MSL)  (Zhan and Li, 2010) | 5.14～6.1 | High-quality, red mid-late maturity variety with medium cold resistance. | Small seed, seed coat is relatively thin. Each fruit contains 1～4 seeds. |
| Cabernet Sauvignon (CS)(Zhang, 2000) | 5.17～6.3 | High-quality, red late-maturing variety with medium cold resistance. | Medium-sized seed, seed coat is medium thickness. Each fruit contains 1～3 seeds. |
