# Supplementary material for: Fertility analysis of intraspecific hybrids in Vitis vinifera and screening of superior hybrid combinations
Source: Front Plant Sci. 2022 Aug 11;13:940540. doi: 10.3389/fpls.2022.940540 (PMC9403984; doi:10.3389/fpls.2022.940540)
Supplement: Supplementary file 1 [file Data_Sheet_1.zip › Supplementary Material/Table 2.docx]

**Table S2 Different crosses and selfing combinations**

| Combinations（♀×♂） | Number | Quantity/spike | | Emasculation date | | Pollination date | | Harvest date | |
| --- | --- | --- | --- | --- | --- | --- | --- | --- | --- |
|  |  | 2020 | 2021 | 2020 | 2021 | 2020 | 2021 | 2020 | 2021 |
| CS×ECL | C1 | 20 (6) | 12 (6) | 5/20 | 5/19 | 5/22 | 5/21 | 9/26 | 10/9 |
| MSL×ML | C2 | 12 (6) | 6 (6) | 5/19 | 5/18 | 5/21 | 5/20 | 9/26 | 10/9 |
| MSL×ECL | C3 | 8 (6) | 20 (6) | 5/19 | 5/18 | 5/21 | 5/20 | 9/26 | 10/9 |
| CS×ML | C4 | 20 (6) | 6 (6) | 5/20 | 5/19 | 5/22 | 5/21 | 9/26 | 10/9 |
| DKF×ML | C5 | 15 (6) | 20 (6) | 5/17 | 5/17 | 5/19 | 5/19 | 9/5 | 9/9 |
| DKF×ECL | C6 | 6 (6) | 6(6) | 5/17 | 5/17 | 5/19 | 5/19 | 9/5 | 9/9 |
| ECL×CS | C7 | 6 (6) | 20 (6) | 5/18 | 5/16 | 5/20 | 5/18 | 9/5 | 9/11 |
| ECL×GN | C8 | 15 (6) | 6 (6) | 5/18 | 5/16 | 5/20 | 5/18 | 9/5 | 9/11 |
| ECL×MSL | C9 | 8 (6) | 6 (6) | 5/18 | 5/16 | 5/20 | 5/18 | 9/5 | 9/11 |
| ECL×DKF | C10 | 6 (6) | 15 (6) | 5/18 | 5/16 | 5/20 | 5/18 | 9/5 | 9/11 |
| ML×GN | C11 | 15 (6) | 10 (6) | 5/16 | 5/14 | 5/18 | 5/16 | 9/23 | 10/7 |
| ML×CS | C12 | 6 (6) | 20 (6) | 5/16 | 5/14 | 5/18 | 5/16 | 9/23 | 10/7 |
| ML×MSL | C13 | 10 (6) | 10 (6) | 5/16 | 5/14 | 5/18 | 5/16 | 9/23 | 10/7 |
| ML×DKF | C14 | 15 (6) | 10 (6) | 5/16 | 5/14 | 5/18 | 5/16 | 9/23 | 10/7 |
| ML | C15 | 6 (6) | 6 (6) | / | / | / | / | 9/23 | 10/7 |
| ECL | C16 | 6 (6) | 6 (6) | / | / | / | / | 9/5 | 9/11 |
| GN | C17 | 6 (6) | 6 (6) | / | / | / | / | 8/27 | 9/5 |
| DKF | C18 | 6 (6) | 6 (6) | / | / | / | / | 9/5 | 9/9 |
| MSL | C19 | 6 (6) | 6 (6) | / | / | / | / | 9/26 | 10/9 |
| CS | C20 | 6 (6) | 6 (6) | / | / | / | / | 9/26 | 10/9 |

Note: ML, ECL, GN, DKF, MSL, and CS represent cultivars of Meili, Ecolly, Garanior, Dunkelfelder, Marselan, and Cabernet Sauvignon. After the first pollination, 3 pollinations were carried out for 3 consecutive days.
