# Supplementary material for: Fertility analysis of intraspecific hybrids in Vitis vinifera and screening of superior hybrid combinations
Source: Front Plant Sci. 2022 Aug 11;13:940540. doi: 10.3389/fpls.2022.940540 (PMC9403984; doi:10.3389/fpls.2022.940540)
Supplement: Supplementary file 1 [file Data_Sheet_1.zip › Supplementary Material/Table 3.docx]

**Table S3 Capacity and weight of fertility indicator of tested combinations**

| Time | 2020 | | | | 2021 | | | |
| --- | --- | --- | --- | --- | --- | --- | --- | --- |
| Principal components | Prin1 | Prin2 | Composite score coefficient | Weight  (%) | Prin1 | Prin2 | Composite score coefficient | Weight  (%) |
| Characteristic root | 2.971 | 1.682 |  |  | 3.403 | 1.191 |  |  |
| Variance interpretation (%) | 49.52 | 28.03 |  |  | 56.71 | 19.8 |  |  |
| FSR | 0.5481 | 0.0143 | 0.3552 | 17.50 | 0.4877 | 0.1543 | 0.4013 | 17.97 |
| Sr | 0.5538 | 0.0583 | 0.3747 | 18.46 | 0.5124 | 0.1455 | 0.4173 | 18.68 |
| CI | 0.5564 | 0.0752 | 0.3824 | 18.85 | 0.4839 | 0.2727 | 0.4291 | 19.21 |
| GR | 0.227 | 0.4573 | 0.3103 | 15.29 | 0.2262 | 0.7096 | 0.3515 | 15.74 |
| ER | 0.1557 | 0.6031 | 0.3174 | 15.64 | 0.4513 | 0.3791 | 0.4326 | 19.37 |
| SR | 0.0872 | 0.6464 | 0.2893 | 14.26 | 0.1031 | 0.4832 | 0.2016 | 9.03 |
